# Supplementary material for: Investigating university English as a foreign language instructors’ implementations in teaching integral listening with speaking
Source: PLoS One. 2025 Aug 8;20(8):e0327029. doi: 10.1371/journal.pone.0327029 (PMC12334060; doi:10.1371/journal.pone.0327029)
Supplement: S1 Appendix — (DOCX) [file pone.0327029.s001.docx]

**S1 Appendix A 1.** Instructors’ Questionnaire

Dear Instructor,

The main purpose of this questionnaire is to collect data for conducting our study entitled “Investigating University English as a foreign language instructors’ implementations in teaching integral listening with speaking”. Therefore, we kindly request that you complete the item for data collection, which might determine the quality of the findings. The responses you give will be kept confidential and anonymous. You are not expected to write your name and personal information.

Thank you in advance for your willingness!

The Researchers

**Directions:** Read each of the statements in the table below that describe the instructors' practices of teaching English language listening skills in integration with speaking skills. Hence, please circle the number that best indicates your choice using the key for items 17-27.

Key: 1 = Never, 2 = Rarely, 3 = Sometimes, 4 = Always, 5 = Often

| No. | Statement | Likert Scale | | | | |
| --- | --- | --- | --- | --- | --- | --- |
| 17 | I continuously practice teaching listening in integration with speaking for effective EFL learning. | 1 | 2 | 3 | 4 | 5 |
| 18 | I use authentic listening materials like audio and video to practice teaching listening skills in integration with speaking skills. | 1 | 2 | 3 | 4 | 5 |
| 19 | I utilize familiar topics to practice teaching listening skills in integration with speaking skills. | 1 | 2 | 3 | 4 | 5 |
| 20 | I motivate learners to reflect on listening lessons to review listening activities to teach listening in integration with speaking skills. | 1 | 2 | 3 | 4 | 5 |
| 21 | I apply the conversational dialogue to teaching listening skills in integration with speaking skills. | 1 | 2 | 3 | 4 | 5 |
| 22 | I provide feedback to learners in teaching listening in integration with speaking skills. | 1 | 2 | 3 | 4 | 5 |
| 23 | I employ linguistic, discourse, pragmatic, etc., information to practice teaching listening skills in integration with speaking skills. | 1 | 2 | 3 | 4 | 5 |
| 24 | I explicitly teach listening skills in integration with speaking skills to develop learners’ listening competencies. | 1 | 2 | 3 | 4 | 5 |
| 25 | I teach listening in integration with speaking skills by implementing task-based instruction, incorporating speaking content into listening lessons. | 1 | 2 | 3 | 4 | 5 |
| 26 | I effectively implement pre-, while, and post-listening stages to teach listening skills in integration with speaking skills.  . | 1 | 2 | 3 | 4 | 5 |
| 27 | I employ techniques like scaffolding note-taking, opinion sharing, etc., to teach listening skills in integration with speaking skills. | 1 | 2 | 3 | 4 | 5 |
