# Supplementary material for: Investigating university English as a foreign language instructors’ implementations in teaching integral listening with speaking
Source: PLoS One. 2025 Aug 8;20(8):e0327029. doi: 10.1371/journal.pone.0327029 (PMC12334060; doi:10.1371/journal.pone.0327029)
Supplement: S2 Appendix — (DOCX) [file pone.0327029.s002.docx]

**S2 Appendix A2.** Interview

Dear Instructor,

The main purpose of this interview is to collect data for our study entitled “Investigating University English as a foreign language instructors’ implementations in teaching integral listening with speaking”. Hence, we kindly invite you to take part in an interview, as we believe that your information is valuable for our study. The information you give me will be kept confidential.

Thank you for your cooperation!

The Researchers

**Instructors’ practices of teaching listening in integration with speaking**

1. Do you practice teaching listening skills in integration with speaking skills? If yes, please mention important techniques to implement listening and speaking integration. If not, why? For what purpose do you integrate? How frequently do you implement listening and speaking in integration?
2. What kinds of listening materials do you use to teach listening in integration with speaking? Authentic or non-authentic? Why? What listening activities do you use to integrate listening and speaking skills?
3. How do you motivate learners to engage in listening lessons in integration with speaking skills?
