# Supplementary material for: Investigating university English as a foreign language instructors’ implementations in teaching integral listening with speaking
Source: PLoS One. 2025 Aug 8;20(8):e0327029. doi: 10.1371/journal.pone.0327029 (PMC12334060; doi:10.1371/journal.pone.0327029)
Supplement: S3 Appendix — (DOCX) [file pone.0327029.s003.docx]

**S3 Appendix A 3.** Students’ Questionnaire

Dear Students,

The purpose of this questionnaire is to collect data concerning the research title “Investigating University English as a foreign language instructors’ implementations in teaching integral listening with speaking”. Therefore, we kindly request that you complete each item of the questionnaire carefully for the effectiveness of data collection. The responses you give will be kept confidential and anonymous. Please note that you are not expected to write your name and personal information.

Thank you in advance!

The Researchers

Directions**:** Read each statement in the table below that describes the purpose of the study, and then circle the number that best indicates your choice using the key for items 1-11 that are measured by using “Never” to “Often”.

Key: 1 = Never, 2 = Rarely, 3 = Sometimes, 4 = Always, 5 = Often

| No | Statement | Likert Scale | | | | |
| --- | --- | --- | --- | --- | --- | --- |
| 1 | My teacher continuously teaches listening in integration with speaking for effective learning. | 1 | 2 | 3 | 4 | 5 |
| 2 | My instructor uses authentic listening materials like audio and video to practice teaching listening skills in integration with speaking skills. | 1 | 2 | 3 | 4 | 5 |
| 3 | My instructor utilizes familiar topics to teach listening skills in integration with speaking skills. | 1 | 2 | 3 | 4 | 5 |
| 4 | My instructor motivates me to reflect on listening lessons and review listening activities to teach listening in integration with speaking skills. | 1 | 2 | 3 | 4 | 5 |
| 5 | My instructor applies the conversational dialogue to teaching listening skills in integration with speaking skills. | 1 | 2 | 3 | 4 | 5 |
| 6 | My instructor provides feedback to me in teaching listening skills in integration with speaking skills. | 1 | 2 | 3 | 4 | 5 |
| 7 | My instructor employs linguistic, discourse, pragmatic, etc. information to practice teaching listening skills in integration with speaking skills. | 1 | 2 | 3 | 4 | 5 |
| 8 | My instructor explicitly (clearly) teaches listening skills in integration with speaking skills to develop my listening competencies. | 1 | 2 | 3 | 4 | 5 |
| 9 | My instructor teaches listening skills in integration with speaking skills by implementing task-based instruction, incorporating speaking content into listening lessons. | 1 | 2 | 3 | 4 | 5 |
| 10 | My instructor effectively implements pre-listening, while, and post-listening stages to teach listening skills in integration with speaking skills.  . | 1 | 2 | 3 | 4 | 5 |
| 11 | My instructor employs techniques like scaffolding note-taking, opinion sharing, etc., to teach listening skills in integration with speaking skills. | 1 | 2 | 3 | 4 | 5 |
