# Supplementary material for: Investigating university English as a foreign language instructors’ implementations in teaching integral listening with speaking
Source: PLoS One. 2025 Aug 8;20(8):e0327029. doi: 10.1371/journal.pone.0327029 (PMC12334060; doi:10.1371/journal.pone.0327029)
Supplement: S4 Appendix — (DOCX) [file pone.0327029.s004.docx]

**S4 Appendix A 4.** Observation checklist

Instructor’s name or Pseudonym: ______________

Classroom: ______________

Course name: ____________

Duration: ______________

Lesson topic: ____________

Date: ______________

**Rating scales**:

1= Not Evident means practice is not implemented;

2 = Rarely Evident means practice is implemented scanty;

3 = Somewhat Evident means practice is minimally implemented; and

4 = Evident means practice is mostly implemented

**How does teaching listening in integration with speaking look?**

| No. |  | Statement | Rating Scale | | | | | |
| --- | --- | --- | --- | --- | --- | --- | --- | --- |
|  |  | **Pre-listening** |  |  |  |  |  |  |
| 1 | Before listening tasks, the teacher asks learners to talk about their experiences on a topic. | | | 1 | 2 | 3 | 4 | 5 |
| 2  3 | The teacher asks general questions or brainstorms about a topic to learners to activate their schemata. | | | 1 | 2 | 3 | 4 | 5 |
| 3 | Before listening tasks, the teacher asks learners to guess unfamiliar words or phrases. | | | 1 | 2 | 3 | 4 | 5 |
| 4 | Before listening, the teacher gets learners’ attention on visuals like diagrams, pictures, charts, etc., based on which learners speak. | | | 1 | 2 | 3 | 4 | 5 |
| 5 | The teacher asks learners to predict vocabulary that they might listen to in the content. | | | 1 | 2 | 3 | 4 | 5 |
|  | While listening | | |  |  |  |  |  |
| 6 | During listening tasks, the teacher asks the learners whether they encounter challenges like knowledge related to grammar and vocabulary. | | | 1 | 2 | 3 | 4 | 5 |
| 7 | The teacher sets up listening tasks and repeats reading for learners’ understanding before their oral production. | | | 1 | 2 | 3 | 4 | 5 |
| 8 | The teacher helps learners to take notes which helps them comprehend listening texts for speech production. | | | 1 | 2 | 3 | 4 | 5 |
| 9 | The teacher pays selective attention to listening tasks to manage learners’ motivation which prepares learners for listening and speaking in integration. | | | 1 | 2 | 3 | 4 | 5 |
| 10 | During listening, the teacher checks learners’ listening comprehension or reviews previous knowledge and lets they speak. | | | 1 | 2 | 3 | 4 | 5 |
| 11 | The teacher lets learners work in pairs or small groups to discuss and understand tasks, and then respond orally. | | | 1 | 2 | 3 | 4 | 5 |
| 12 | The teacher encourages learners to share their opinions to teach listening and increase oral communication. | | | 1 | 2 | 3 | 4 | 5 |
| 13 | The teacher provides clear instructions and a description of tasks for effective oral communication. | | | 1 | 2 | 3 | 4 | 5 |
| 14 | The teacher asks learners to verify their prediction in pre-listening. | | | 1 | 2 | 3 | 4 | 5 |
| 15 | Learners actively engage in integrated aural-oral EFL classes. | | | 1 | 2 | 3 | 4 | 5 |
|  | Post-listening | | |  |  |  |  |  |
| 16 | The teacher asks the learners to give an oral summary of listening tasks. | | | 1 | 2 | 3 | 4 | 5 |
| 17 | After listening, the teacher asks learners to reflect on task difficulty to improve their listening and speaking ability. | | | 1 | 2 | 3 | 4 | 5 |
| 18 | The teacher gives time to learners to evaluate their learning or listening comprehension. | | | 1 | 2 | 3 | 4 | 5 |
| 19 | The teacher lets learners ask questions orally after listening tasks for details. | | | 1 | 2 | 3 | 4 | 5 |
|  | Input for teaching listening integrated with speaking | | |  |  |  |  |  |
| 20 | The teachers use audio-visual (authentic material) to enhance teaching listening integrated with speaking. | | | 1 | 2 | 3 | 4 | 5 |
| 21 | The teacher read aloud listening texts to be heard by learners during teaching listening as integrated with speaking. | | | 1 | 2 | 3 | 4 | 5 |
| 22 | The teacher uses familiar and interesting topics to teach listening in integration with speaking. | | | 1 | 2 | 3 | 4 | 5 |
| 23 | Instructors use conversational dialogues to teach listening in integration with speaking. | | | 1 | 2 | 3 | 4 | 5 |
| 24 | The nature of listening tasks integrates speaking in the classroom. | | | 1 | 2 | 3 | 4 | 5 |

**Classroom observation (open-ended)**

- implementation of listening phases:
  - pre-listening
  - while listening
  - post-listening
- the nature of teaching material
- authentic
- non-authentic
- techniques for teaching aura-oral skills
- read aloud texts
- conversation dialogue
- characteristics of listening topics
- familiar
- unfamiliar
