# Supplementary material for: Investigating university English as a foreign language instructors’ implementations in teaching integral listening with speaking
Source: PLoS One. 2025 Aug 8;20(8):e0327029. doi: 10.1371/journal.pone.0327029 (PMC12334060; doi:10.1371/journal.pone.0327029)
Supplement: S5 Appendix — (DOCX) [file pone.0327029.s005.docx]

**S5.Appendix A 5.** The three-cycle coding methods summarized

FIRST CYCLE CODING

In Vivo Coding (direct language of participants), Initial Coding, and/or Values Coding (for interview transcripts as a method of attuning yourself to participant language, perspectives, and worldviews)

SECOND CYCLE CODING

1. Eclectic Coding (for refining your First Cycle choices)

2. Pattern Coding and/or Focused Coding (for categorization of your coded data as an initial analytic strategy)

AFTER SECOND CYCLE CODING [THIRD CYCLE]

Organizing themes for pre-writing and data analysis and beginning interpretations

In this phase a researcher ask hem/herself questions like: “Have I got it right?” [Our study thoroughly revised and checked all children noes], “Did I learn anything new?” [Our study refined emerging codes], and “Now what do I do?” [The present study carefully analyzed data based on thematically coded categories].

(Adapted from: Saldaña, 2021). Coding techniques for quantitative and mixed data.
