# Supplementary material for: Investigating university English as a foreign language instructors’ implementations in teaching integral listening with speaking
Source: PLoS One. 2025 Aug 8;20(8):e0327029. doi: 10.1371/journal.pone.0327029 (PMC12334060; doi:10.1371/journal.pone.0327029)
Supplement: S6 Appendix — (DOCX) [file pone.0327029.s006.docx]

**S6.Appendix A 6.** Interview transcription

**INTERVIEWS**

**Audio file: T1**[**.mp3**](https://wsu12-my.sharepoint.com/personal/addisu_bogale_wsu_edu_et/Documents/Transcribed%20Files/01.%20Dr.%20Abate%201.mp3)**[11 minutes]**

**Transcript**

**Instructors’ practices of teaching listening in integration with speaking**

**Researcher**: Do you integrate teaching listening skills with speaking skills? If yes, please mention important techniques. If not, why? For what purpose do you integrate? How frequently do you practice listening and speaking in integration?

**Teacher 1**: It's very difficult to say I integrate it, but though it's not intentional, whenever I teach listening skills, I just provide some chance for students to reflect on what they listen. So this is, I think, integration, and this is the practice; there is no explicit instruction is not there.  I can say that because listening and speaking parts are there, my focus most of the time is to practice giving some chance, not intentionally, but it is by default. The material by itself requires integrating these skills, but I do not do it intentionally. Yet, it can be taken as intentional integration can be taken because the material is there, and our students have to reflect on these after listening.

It is very difficult to say to this degree or this level, but I can say that the integration is not to the expected level, but is supposed to support each other, and the integration is not taking place.

**Researcher**: What kinds of listening materials do you use to teach listening skills in integration with speaking skills? Adapted or adopted? Why?

**Teacher 1**: Yes. I adapt. What is already there and what I find through different sources and use in the classroom. Thus, I think it is important when doing that. This is because it gives me some room to understand my students and to bring that material to the level of my students and support them. You know, students are of different level of different levels of understanding. So just mix up activities.

**Researcher**: What listening activities do you use to integrate listening skills with speaking skills?

**Teacher 1**: Yeah, the activities are different. For example, I just use audio-recorded material for students to listen to and design. I ask the whole class to reflect on what they listen to, and sometimes I call upon a single student to come to the front and say something about what he or she reflects on. Using this is one way of just doing or practicing these skills in integration. The other I just prepared the presentation topic for the next session presentation.

**Researcher**: How do you motivate learners in listening lessons in integration with speaking skills?

**Teacher 1**: I just put them in groups and ask them to reflect on listening or speaking. For example, if the activity is speaking, if somebody presents then I give chances to discuss together, and then give some kind of assessment in the group that might be out of 10 and like that. So, students could be engaged in each activity and they just actively participate in the group.

**Audio file:** [**T2.mp3**](https://wsu12-my.sharepoint.com/personal/addisu_bogale_wsu_edu_et/Documents/Transcribed%20Files/02.%20Dr.%20Adanech.mp3)**[17’**]

**Transcript**

**Instructors’ practices of teaching listening in integration with speaking**

**Researcher**: Do you integrate teaching listening skills with speaking skills? If yes, please mention important techniques. If not, why? For what purpose do you integrate? How frequently do you practice listening and speaking in integration?

**Teacher 2**: I record my voice and share it with the students to learn my accent, specifically for some topic. I give my record, and for other topics, I give them the native speakers. There are some native speakers' recorded materials, and then activities that they try to practice in communicative activities. We have a huge language laboratory where there are desktops, speakers, have all the necessary materials for listening, but not being used effectively for implementation.

Just students listen and I check their comprehension, otherwise, how can we know their understanding? I check the meaning and the pronunciation at the same time. They provide answers based on the listening lessons. They provide audio for them, and I've observed from the literature that has to be maybe 6 minutes of audio is important, not more than that. If we have some new words that are new for the students, whether they understood that one, I check their comprehension. I gather their answers and compare how they answered through speech, giving a summary of the audio. They achieved the level required in this thing, so I tried my best as an experienced teacher.

**Researcher**: What kinds of listening materials do you use to teach listening skills in integration with speaking skills? Adapted or adopted? Why?

**Teacher 2**: I adapt it and use it.

**Researcher**: **Researcher**: What listening activities do you use to integrate listening skills with speaking skills?

**Teacher 2**: Yes, as I already mentioned, specifically summarization and vocabulary, so that they understood. I check them, they speak out. Some points of main ideas, or speak about the main idea, specific idea, and sometimes I give a chance to evaluate the speaker itself like me. I ask learners for comprehension, i.e., the comprehension questions.

**Researcher**: How do you motivate learners to engage in listening lessons in integration with speaking skills?

**Teacher 2**: This is what we always do. A language teacher has to engage learners. Many teachers have different ways to engage learners. Every teacher, specifically a language teacher, has to do that [engage students]. If teachers talk more, learners have no way to give feedback or learn based on the speaking they have or whatever they listen to. They need to have chances. Yeah! The classroom needs to be student-centered. I give gaps to ask questions to speak after listening. You have to decide whether students to learn in the listening lessons. In that way, I help them to learn something. Therefore, they engage in listening to me and give them chances to ask questions and answer them because engaging learners is very important.

You balance talking since teaching in the university is very tough, unlike high school. We have two to three hours given to teach the Communicative English Language Skills course, which might not be enough, but you give students a chance to speak and balance your talk in the classroom. Unless you [negatively] influence learning the language

**Audio file:** [**T3.mp3**](https://wsu12-my.sharepoint.com/personal/addisu_bogale_wsu_edu_et/Documents/Transcribed%20Files/03.%20Dr.%20Samuel%20Interview.mp3)**(22’)**

**Transcript**

**Instructors’ practices of teaching listening in integration with speaking**

**Researcher**: Do you integrate teaching listening skills with speaking skills? If yes, please mention important techniques. If not, why? For what purpose do you integrate? How frequently do you practice listening and speaking in integration?

**Teacher 3**: It is difficult to say that skills should be integrated, but I try my best to practice, as technology and civilization are there to use. It is better to bring the focus and mix these skills.

**Researcher**: What kinds of listening materials do you use to teach listening skills in integration with speaking skills? Adapted or adopted? Why?

**Teacher 3**: I adapt it and use different materials. I do not use the existing material already given. It helps to provide additional material to benefit them. I can say I try to integrate it, but that is not enough.

**Researcher**: What kinds of listening activities do you use to integrate listening skills with speaking skills?

**Teacher 3**: The common one is the lecture method, but I don’t know if the focus should be testing knowledge or skills, or meaning, that is our problem and approach to teaching. This is the problem, teachers. There is a problem for teachers, including me. The main context should be context, conversation, and telephoning in their group. Another thing is that you give them much description, narration, radio, and television, and they can listen and speak. I read the story to them and spoke accordingly.

**Researcher**: How do you motivate learners to engage in listening lessons in integration with speaking skills?

**Teacher 3**: I give some practice activities to speak out, and some of them listen. And others take the turn in this way, I check their understanding to develop their listening. it is good to give more chances to listening and speaking.

In my observation, students are not trying to practice independently; sometimes, they lose social experience and those things. Nowadays, students are not effective; the problem is not only with them but might be with teachers, and the teaching approach should be researched.

**Audio file: T4**[**.mp3**](https://wsu12-my.sharepoint.com/personal/addisu_bogale_wsu_edu_et/Documents/Transcribed%20Files/04.%20Dr.%20Tsegaberhan.mp3)**(17’)**

**Transcript**

**Instructors’ practices of teaching listening in integration with speaking**

**Researcher**: Do you integrate teaching listening skills with speaking skills? If yes, please mention important techniques. If not, why? For what purpose do you integrate? How frequently do you practice listening and speaking in integration?

**Teacher 4**: As much as possible, I try to teach these skills and try to use the communicative approach in the classrooms. I do invite students to participate and try to integrate these skills.

**Researcher**: What kinds of listening materials do you use to teach listening skills in integration with speaking skills? Adapted or adopted? Why?

**Teacher 4**: I try to contextualize the material to make it easily understandable, try to bring commonly known materials, and adapt, but sometimes I adopt it. I do not think the materials for my students make it easy to make it understand

**Researcher**: What kinds of listening activities do you use to integrate listening skills with speaking skills?

**Teacher 4**: Most of the time, I provide group activities followed by individual activities. I use dialogue and conversation activities.

**Researcher**: How do you motivate learners to engage in listening lessons in integration with speaking skills?

**Teacher 4**: As far as my 20 years at university are concerned, I have enough experience, so as much as possible, I try to engage students in the classrooms. I motivate them, encourage them, and activate them; I hope my teaching classes are interactive. I let them respond to activities and let them participate actively.

**Audio file:** [**T5.mp3**](https://wsu12-my.sharepoint.com/personal/addisu_bogale_wsu_edu_et/Documents/Transcribed%20Files/05.%20Mr.%20Abdulselam.mp3) **(35’)**

**Transcript**

**Instructors’ practices of teaching listening in integration with speaking**

**Researcher**: Do you integrate teaching listening skills with speaking skills? If yes, please mention important techniques. If not, why? For what purpose do you integrate? How frequently do you practice listening and speaking in integration?

**Teacher 5**: When we see the practicality, simply standing in front of students is not a practice rather balancing each phase. Pre, while, and post-listening phases do not focus on balanced activities. Listening skills are ignored like speaking skills, not only at the high school level but also at the university level, as I have observed.

I implement listening phases like pre-, while, and post-listening, but in reality, it is difficult to apply as listening is just an ignored skill, and no attention is given as the material is not suitable, and I simply try to use some other related activities.

**Researcher**: What kinds of listening materials do you use to teach listening skills in integration with speaking skills? Adapted or adopted? Why?

**Teacher 5**: I adapt material from YouTube and native speaker voices. VOA English is also important, like short audio, first listen to it carefully, and prepare a short text audio for 6 minutes, and then I will bring activities for students. This is because the EFL and the native speakers’ accents are quite different.

**Researcher**: What listening activities do you use to integrate listening skills with speaking skills?

**Teacher 5**: I use the communicative type of activities to teach listening and speaking together. I have to critically observe the text and come up with activities based on the listening phases. I use small group discussions, based on the text, but classes are not suitable for frequently practicing these skills.

**Researcher**: How do you motivate learners to engage in listening lessons in integration with speaking skills?

**Teacher 5**: I use a small group discussion, but most of the time, I engage them in a whole class. I teach the techniques of note-taking before speaking. I give extra audio for practice, not the target audio, but my target is text reading aloud rather than audio.

**Audio file: T6.** [**mp3**](https://wsu12-my.sharepoint.com/personal/addisu_bogale_wsu_edu_et/Documents/Transcribed%20Files/06.%20Mr.%20Deguale%20Mk..mp3) **(16’)**

**Transcript**

**Instructors’ practices of teaching listening in integration with speaking**

**Researcher**: Do you integrate teaching listening skills with speaking skills? If yes, please mention important techniques. If not, why? For what purpose do you integrate? How frequently do you practice listening and speaking in integration?

**Teacher 6**: Yeah. Naturally, they are integrated. When we speak, someone is there to listen. So, I practice the lesson in a way that students integrate these skills, but I do not intentionally teach these skills. Nowadays, I have a limited number sometimes I do, but if I get large classes, I try to do that.

**Researcher**: What kinds of listening materials do you use to teach listening in integration with speaking? Adapted or adopted? Why?

**Teacher 6**: The solution could be that the design material has to be designed in a way to entertain both listening and speaking. The number of students should be, but mostly their number is about 50 or above. Both skills of listening and speaking skills should be appropriate. Otherwise, it will be difficult to cope with. Teachers are expected to prepare their materials thus, I adapt the material.

**Researcher**: What kinds of listening activities do you use to integrate listening skills with speaking skills?

**Teacher 6**: Yeah, I mostly present the tasks, activities, the listening activities that are familiar there for the students, especially sports and such interesting social activities. Then I give them the question that they're going to answer and tell them to listen. So these are the types of strategies I follow that will be interesting.

**Researcher**: How do you motivate learners to engage in listening lessons in integration with speaking skills?

**Teacher 6**: Ok, when we come to the speaking section, I want and let them speak individually as well as in a group. I provide listening tasks and give them time to speak based on the listening input.

**Audio file:** [**mp3**](https://wsu12-my.sharepoint.com/personal/addisu_bogale_wsu_edu_et/Documents/Transcribed%20Files/07.%20Mr.%20Elias%20K..mp3) **T7 (21’)**

**Transcript**

**Instructors’ practices of teaching listening in integration with speaking**

**Researcher**: Do you integrate teaching listening skills with speaking skills? If yes, please mention important techniques. If not, why? For what purpose do you integrate? How frequently do you practice listening and speaking in integration?

**Teacher 7**: I didn't do it intentionally to teach listening because listening is somewhat difficult for the learners. They are not happy to learn to listen because of their poor background, or it is because of their hatred of the course. Sometimes, not only I but also most teachers skip the listening section. This is why listening is a challenging task for both teachers as well as students. If you intend to deliver a good listening lesson, it takes time and effort. If you want to do it in such a manner, the implementation can be effective. Therefore, sometimes I skip teaching listening lessons because of the lack of effective listening tasks, guides, as well as any other recorded resources, that are why the emphasis on listening is below the expected level.

It is better if I use listening and speaking skills in an integrated manner, so that, in my experience, I teach listening first and then give tasks to students for students to check their understanding. This needs to group them into a small group to learn the language, listening, and speaking skills in the classroom. To know the general message of the text, I usually did an oral summary of the text after listening to evaluate their understanding. I also give feedback if they miss or misunderstand the listening activities through speaking.

**Researcher**: What kinds of listening materials do you use to teach listening in integration with speaking? Adapted or adopted? Why?

**Teacher 7**: The scope of the material is not conducive to teaching skills in an integrated manner in the classroom. I sometimes use some materials from other materials to make good listeners rather than depending on the existing material. If the material is not appropriate, I will adapt the material. I don't think so. The material only sets the main contents or a title and needs to do some more appropriate tasks that help us to develop learners' learning skills.

My level of degree to accept this degree is below average because the material is not appropriate to teach, and also other contents, and the skills this course.

**Researcher**: What kinds of listening activities do you use to integrate listening skills with speaking skills?

**Teacher 7**: Some activities are repeatedly stated there, but for some skills, there is a scarcity of tasks for students because the material is prepared not in a way that it needs appropriate content of these skills, or some important points are missed.

**Researcher**: How do you motivate learners to engage in listening lessons in integration with speaking skills?

**Teacher 7**: I motivate them more by providing different tasks during listening; I motivate them with note-taking, matching, and so on. Listening and registering, listening and answering questions. This should be told first before they will begin to listen to the text, and when I read the material for the second time, and for the last time. I provide a variety of task questions that engage students in the classroom.

I let students make their groups into pairs or three to discuss the main issue about the listening lessons. Based on this, they can share their understanding with other students in different groups. Some activities that can be filled by them are provided for the class discussion, and finally summarized orally

**Audio file:** [**T8.**](https://wsu12-my.sharepoint.com/personal/addisu_bogale_wsu_edu_et/Documents/Transcribed%20Files/08.%20Mr.%20Zewde%20Tura.mp3)**mp3 (29’)**

**Transcript**

**Instructors’ practices of teaching listening in integration with speaking**

**Researcher**: Do you integrate teaching listening skills with speaking skills? If yes, please mention important techniques. If not, why? For what purpose do you integrate? How frequently do you practice listening and speaking in integration?

**Teacher 8**: Sometimes I try to just download the audio or video from YouTube. I let learners learn, but it is not satisfying. This indicates my belief in teaching these skills.

**Researcher**: What kinds of listening materials do you use to teach listening in integration with speaking? Adapted or adopted? Why?

**Teacher 8**: To make standard material, it should be a consistent and balanced approach, thus, some teachers are not eager to teach these skills, which allows them to skip the listening section. I do not plan, but I unconsciously read the material and just give a chance to students, I give lectures, and then I ask them. How can they get from lectures to listening lessons? It is difficult to teach in this way these skills actually in the classrooms. In this way, they cannot effectively practice listening and then speak out.

**Researcher**: What kinds of listening activities do you use to integrate listening skills with speaking skills?

**Teacher 8**: I use some technique. I pick out some words from the listening text, prepare some questions, and let them read the text first to fill in gaps, true-false questions, and get some main idea from the text by playing audio or reading twice or more. This is to test their knowledge rather than teach the language or the meaning of the text. This is difficult to learn these skills. It needs individual practices.

**Researcher**: How do you motivate learners to engage in listening lessons in integration with speaking skills?

**Teacher 8**: Listening skills are personal and individual work, but you let them listen individually and reflect on the material about the text in pairs or groups. However, listening is a very natural individual issue, and the level of understanding is also different. Therefore, it is a challenging task to teach listening and speaking skills in actual classroom practice.

**Audio file: T9**[**.mp3**](https://wsu12-my.sharepoint.com/personal/addisu_bogale_wsu_edu_et/Documents/Transcribed%20Files/09.%20%20Dr.%20Abebe%20L.,%20WCU.mp3)**(17’)**

**Transcript**

**Instructors’ practices of teaching listening in integration with speaking**

**Researcher**: Do you integrate teaching listening skills with speaking skills? If yes, please mention important techniques. If not, why? For what purpose do you integrate? How frequently do you practice listening and speaking in integration?

**Teacher 9**: Sure, that means I struggle to teach in an integrated manner. OK, I believe that teaching all the skills in an intermingled manner is very important. The purpose maybe I need to determine some specific information about what they listen to. I may expect my students to identify the gist (the main idea) of what they are listening to determine some specific idea.

**Researcher**: What kinds of listening materials do you use to teach listening in integration with speaking? Adapted or adopted? Why?

**Teacher 9**: For example, you have to relate the tasks, the activities where your students' level of understanding, as well as students' background information. Unless, for example, you are talking about the country of America, they may not know about it, and sports, the economy, and some issues of Ethiopia, particularly in this area, they can easily understand. That's why I adapted the material to make, modify, modify, and capture the attention of Ethiopia. If you bring the material related to Ethiopia, you motivate and easily make them interested in listening rather than depending on the existing material. If you are talking about the culture of other aspects of another continent, they ignore you.

**Researcher**: What kinds of listening activities do you use to integrate listening skills with speaking skills?

**Teacher 9**: Actually, I prepare the content. The content may be related to students’ background information; the task should be related to their educational background and may be related to some contemporary issues. I arrange these activities to listen based on the purpose, i.e., mostly communicative activities. You let them listen, then you ask them to write what they have listened to, and I expect them to reflect on what they have listened to, jot down, and speak accordingly.

**Researcher**: How do you motivate learners to engage in listening lessons in integration with speaking skills?

**Teacher 9**: I use different ways to motivate them, though listening is an individual activity. I ordered them to listen individually and jot down individually, then I needed them to share what they listened to with others (opinion sharing). I encourage them to share with others. Finally, by selecting one individual, I let them reflect on what they agreed. You may also encourage your lenders to list, practice jotting down, and then reflect individually. After this, I may encourage reflection in written form, not orally. If you want to evaluate individually, you give them individual activities rather than in groups, and make them reflect. And I provide feedback to correct or not.

**Audio file: T10.**[**mp3**](https://wsu12-my.sharepoint.com/personal/addisu_bogale_wsu_edu_et/Documents/Transcribed%20Files/10.%20Dr.%20Aman%20T.,%20WCU.mp3) **(22’)**

**Transcript**

**Instructors’ practices of teaching listening in integration with speaking**

**Researcher**: Do you integrate teaching listening skills with speaking skills? If yes, please mention important techniques. If not, why? For what purpose do you integrate? How frequently do you practice listening and speaking in integration?

**Teacher 10**: When I was teaching the course on listening skills for the previous classes, I always utilized some of the activities that are meant for speaking, and so I always try to just integrate these skills. However, I didn't practice for the course of Communicative English Language but other courses like major courses, which were designed for first or second-year students, like spoken English and advanced language skills; I utilized those activities and integrated skills. This motivates students, and they actively participate in the classroom.

**Researcher**: What kinds of listening materials do you use to teaching listening in integration with speaking? Adapted or adopted? Why?

**Teacher 10**: Yeah. I adapt the material because if the material is not encouraging activities that are good for classroom teaching, I try to adapt based on students' curiosity, which interests them. I design activities for the students so I can easily adapt the material based on the students’ culture that the activity or the topic is chosen helps them.

**Researcher**: What kinds of listening activities do you use to integrate listening skills with speaking skills?

**Teacher 10**: Yeah. I sometimes use both explicit and implicit. As a teacher, you always know what to do with the course. I try to make activities realistic and to be realistic. So, I sometimes plan it. Sometimes I come up with the integration of related skills, listening, and speaking. I just choose some activities in a planned manner. Particularly, in this course, I didn’t practice because it is not good to effectively practice skills in an integrated way, and no effective activities are there material, and it is ignored. I use some of the activities like dialogue completion, repetitive drills, filling gaps, dictation, and note-taking, which are helpful to integrate listening and speaking skills in the language classes.

**Researcher**: How do you motivate learners to engage in listening lessons in integration with speaking skills?

**Teacher 10**: Yeah. Students should be engaged in activities of listening and speaking as they first listen and should be engaged through speaking, but listening by its nature is an individual activity. For speaking, listening is an input that they should be engaged and should be independent learner and practice outside the classroom, like multimedia, and it should be based on their effort. This increases their comprehensible input and output should be there, so that EFL learners are highly encouraged and actively participate as both listening and speaking are highly integrated skills.

**Audio file: T11.**[**mp3**](https://wsu12-my.sharepoint.com/personal/addisu_bogale_wsu_edu_et/Documents/Transcribed%20Files/11.%20Mr.%20Ermias,%20WCU.mp3) **(28’)**

**Transcript**

**Instructors’ practices of teaching listening in integration with speaking**

**Researcher**: Do you integrate teaching listening skills with speaking skills? If yes, please mention important techniques. If not, why? For what purpose do you integrate? How frequently do you practice listening and speaking in integration?

**Teacher 11**: This is most of the time our problem, and we do it unplanned way. By its nature, listening is dependent on speaking and needs a planned way. I sometimes do this internally. I sometimes ignore this thing because of some limitations, as mentioned before. However, I don’t do it intentionally.

I try to select some students in the classroom, pick them up, and let them respond to some questions orally. If they give some responses, I check their understanding using speaking and try to summarize or paraphrase the main points in the classroom, remembering some major points of the lesson.

**Researcher**: What kinds of listening materials do you use to teach listening in integration with speaking? Adapted or adopted? Why?

**Teacher 11**: Currently, I am using the existing material, but we are preparing material to fill the gaps. I am trying, but not sufficient. We have different types of smartphones that we can access video or audio, but there is a gap in this area, referring to different literature, and then merging them and giving them to students to discuss.

**Researcher**: What kinds of listening activities do you use to integrate listening skills with speaking skills?

**Teacher 11**: I do have tasks for discussions in groups, lecturing them, through which I let them discuss and check understanding. I also use presentation techniques on some points, and to evaluate their listening abilities. I use dialogue, conversations, etc. I evaluate the use of these techniques.

**Researcher**: How do you motivate learners to engage in listening lessons in integration with speaking skills?

**Teacher 11**: I motivate them. They can understand if I try to motivate, and pay attention, sometimes audio material, though I lack. I let them practice individually and create their training or practice, as some of them have a good concept about listening strategies of listening but the problem is how they practice in the classroom.

**Audio file: T12.**[**mp3**](https://wsu12-my.sharepoint.com/personal/addisu_bogale_wsu_edu_et/Documents/Transcribed%20Files/12.%20Dr.%20Mesfin%20M.1.mp3)**(23’)**

**Transcript**

**Instructors’ practices of teaching listening in integration with speaking**

**Researcher**: Do you integrate teaching listening skills with speaking skills? If yes, please mention important techniques. If not, why? For what purpose do you integrate? How frequently do you practice listening and speaking in integration?

**Teacher 12**: I may not say that I practice according to the principle of integrated skills, but I try to teach how it is designed to teach. We have already prepared material, and we are guided only by using it rather than effectively integrating it in a principled manner. I think these skills, as a general rule, should be integrated, but speaking demands listening. We may focus on one skill, like writing or speaking, but not explicitly integrate it; rather, I implicitly integrate it or not in a planned manner, believing that these skills are integrated naturally.

**Researcher**: What kinds of listening materials do you use to teach listening in integration with speaking? Adapted or adopted? Why?

**Teacher 12**: Yeah! I think. Language teaching needs additional teaching materials. Especially to make it practice, students need to practice, as the technology doesn’t allow for adaptation, but I believe in adapting the material. As the material is not appropriate, I sometimes adapt to achieve the objective of the course, though it is not enough.

**Researcher**: What listening activities do you use to integrate listening skills with speaking skills?

**Teacher 12**: To teach these skills in integration, activities are very important. What I do is communicative activities, like role play, dialogue, and conversations. This needs grouping students and using different speaking tasks in their groups. So, I form group work and pair work in the class.

**Researcher**: How do you motivate learners to engage in listening lessons in integration with speaking skills?

**Teacher 12**: Ok, just to integrate, it needs students' active participation. I try to involve them. So, some of the ways used in the classroom, communicative, are one to do I have to group them and vary tasks in the group, and that requires individual work, pair work, and so on, involving conversations. However, teaching each individual in listening and speaking is challenging to teach independently. It is impossible to treat individuals fairly, we have no language lab and other sources to practice, so it is not practical in the classroom.
