# Supplementary material for: Investigating university English as a foreign language instructors’ implementations in teaching integral listening with speaking
Source: PLoS One. 2025 Aug 8;20(8):e0327029. doi: 10.1371/journal.pone.0327029 (PMC12334060; doi:10.1371/journal.pone.0327029)
Supplement: Attachment — Submitted filename: RESPONSES_ Reviewers Comments_PLOS ONE.docx [file pone.0327029.s008.docx]

**Manuscript Revision (PONE-D-24-53961)**

**Response to Reviewer 1**

Thank you so much for your valuable comments you provided for our manuscript to improve its quality for consideration for publication. In the following section.

**Comment (general):** Technical Soundness & Data Support for Conclusions (Partly)
The manuscript presents a valuable study on integrating listening and speaking skills in Ethiopian EFL classrooms. The mixed-methods approach (surveys, interviews, classroom observations) provides insightful findings, but several limitations weaken the strength of the conclusions:

**Response**: Yes, these limitations can affect the quality of the conclusion, thus, we seriously considered all your comments for betterment.

**Comment 1:** Mismatch between Instructor Perceptions and Student Feedback:
Instructors report high integration of listening and speaking skills, but students' survey responses and classroom observations contradict this.
This suggests possible overestimation or bias in self-reported data.
Recommendation: The study should explicitly acknowledge this discrepancy and provide further validation, such as external assessments.

**Response**: Thank you so much for your priceless insight. We accepted your comment and acknowledged it explicitly (see the discussion section, line numbers “829-832”, and p.39).

**Comment 2:** **Lack of Longitudinal Data or Experimental Control:**
The study does not track improvements in students’ listening/speaking skills over time.
No control group is used to compare different instructional strategies.
Recommendation: Future research should consider pre-/post-assessments or comparative studies to strengthen causal claims.

**Response**: We agree with your valuable comment. Thank you. We have recommended and included in limitations (see line numbers “941-942”, p.43).

**Comment 3:** Limited Generalizability:
The study focuses on only two Ethiopian universities, making broader applicability uncertain.
Recommendation: The authors should discuss this limitation and suggest cross-institutional studies for future work.

**Response**: Yes, exactly, we accepted it as is and incorporated limitations and recommended it for further research.

**Comment 4:** 2. Statistical Analysis (Yes)
The statistical analysis is rigorous and appropriate, employing:
Descriptive statistics (means, standard deviations) for survey responses.
Multiple regression analysis to examine the relationship between instructional practices and skill integration.
Normality checks and collinearity diagnostics (VIF & Tolerance).
However, areas for improvement include:
No comparative tests (e.g., t-tests, ANOVA) to examine group differences.
No statistical validation of actual student improvement (e.g., pre/post-tests).
Recommendation: Including comparative analysis and pre/post performance data would enhance empirical validation.

**Response**: Thank you so much for your comment in this regard. We have revised the manuscript considering your comment into account. The revision suggested and acknowledged the limitations (see line number “940”, p. 43).

**Comment 5: 3. Data Availability (No)**
The full dataset (survey responses, coded qualitative data, regression outputs) is not publicly available.
Interview transcripts and NVIVO-coded themes are not included.

Recommendation:
The authors should deposit anonymized raw data in a public repository (e.g., Figshare, Zenodo, Dryad).
If privacy concerns exist, structured data summaries should be provided as supplementary materials.

**Response**: Thank you for showing concern about the guideline, which is very important. Thus, we have uploaded these datasets

**Comment 6:** 5. **Additional Concerns and Recommendations**
Ethical Considerations: The study states that ethical approval was obtained, but more clarity on informed consent procedures (especially for student participants) would be beneficial.

**Response**: Thank you. We revised based on your comment (see line numbers “303-305”, p.13).

**Comment 7:** Study Contribution: The research provides valuable insights into EFL teaching challenges in Ethiopian universities, making it relevant to curriculum developers, instructors, and policymakers.
Recommendation for Strengthening Findings:
Explicitly acknowledge biases in self-reported data.
Use additional data sources for validation (e.g., rubric-based speaking assessments).
Include more structured comparisons with prior research.

**Response**: We appreciate your comment. This is significant for further study as it was not included in the first submission (see line numbers “937-939”, p.43). Limitations were acknowledged (see line numbers “981-987”, p.45).

Language issues, clarity, and structure were seriously considered in revision.

**Manuscript Revision (PONE-D-24-53961)**

**Reviewer 2 Comments and Response to Comments**

First of all authors are thankful and appreciate your precious time you exerted to read our manuscript to shape it. This would help improve our work, move one step ahead before considering it for publication. You provided priceless input for us to thoroughly revise the manuscript. Thank you so much. We tried our best accordingly in the following section.

**Comment 1:** Overall Assessment

This manuscript examines how university EFL instructors implement integral teaching of listening and speaking, a crucial area in applied linguistics. However, the paper contains significant methodological flaws, imprecise language, inadequate engagement with recent literature, and problematic statistical analyses. There are also major inconsistencies between the research objectives, findings, and conclusions. Without substantial revisions, the manuscript does not meet the standard for publication.

**Response**: Thank you so much for this insight. Yes, you are right because these issues should be considered for the quality of the manuscript, though we have missed. We understood the need for critical revision to enhance the quality of the manuscript. Accordingly, we made substantial revisions in each section based on your feedback. For example, in the methodology section, we make it rigorous to improve this section. Likewise, we thoroughly read it and edited the language for accuracy. In addition, we utilized Grammarly to enhance the quality; recent literature was incorporated, and so on.

**Comment 2:** Major Language and Structural Issues (By Section & Page Number)
Abstract (Page 1)
•The abstract is wordy and lacks precision. The phrase “scarce studies in the area” should be replaced with “limited prior research.”
•The sentence “Students’ data confirmed these findings” lacks clarity. Which specific data points confirm the findings? Be explicit.

**Response**: Thank you. We revised most sections to minimize wordiness. We replaced specific phrases as per your comment (see p.2, lines 28-29).

**Comment 3:** •Typos:
•“forwarded recommendations” → should be “provided recommendations.”
•“target variables that improve integral instruction” → should be “key factors influencing integrated instruction.”

**Response**: Thank you. Authors revised it accordingly, please see line numbers 40-41 and 45-46 on p.2. (Abstract section).

**Introduction (Pages 2-4)**

**Comment 4:** •The introduction lacks a clear problem statement. It vaguely suggests that integrated teaching is underutilized without presenting quantifiable evidence or literature-based justification.

**Response:** We appreciate the reviewer's valuable comment. Accepting this idea, we almost changed the introduction section. Particularly, stating or contextualizing the problem statement in line numbers ” 85-135”, in addition to being indicated in the literature section. Hopefully, it makes sense compared to our work before your comment. Your comment better shaped our introduction.

**Comment 5**: •Overuse of generic phrases:
•“Speaking and listening are significant skills for learning other English language skills.” → This is redundant; revise to “Speaking and listening are foundational for English language acquisition.”

**Response**: Yes, you are right. We corrected this comment, more sound than ours. Terminology should be consistent in our study (see line 71 on p.3). The suggested reference in the introduction section for help skills integration is incorporated (L96 and 100, [22] p4).

**Methodology (Pages 5-7)**
•Sampling Issues:
**Comment 6**: •The study claims to have used “systematic sampling” for student participants, but does not explain the criteria for inclusion/exclusion.
•The total sample size of **252 participants** is mentioned, but how were these numbers determined? Was a power analysis conducted?

**Response**: Your comment sounds, and we agree. Thank you so much. Therefore, we have revised sample procedures to include sample size as we did not incorporate the edited copy and the later corrected version, which was our mistake. Considering your comment, we indicated revision from line numbers “358-445, 16…19.” Additionally, the total sample size of “252” is the combined total of teachers (n = 92) and students (n =160), and the determination process is indicated in these lines and pages. Power analysis was not conducted, and we have corrected it in the revision. Procedures were shown to determine the sample size.

**Data Collection Methods**:

**Comment 7**: •The manuscript states that “observations were structured”, but there is no mention of an observation rubric or inter-rater reliability measures.

**Response**: Thank you, dear reviewer, for this comment. In our study, only one observer (principal investigator, currently PhD candidate) was involved, thus, no inter-rater reliability was considered. Instead, we explained to use of PLATO observation scales as observation carried out by the first authors (PhD candidate) rather than observed by different observers or two more raters rate behaviors, but in this study, we have counted the frequency and numbered it, then interpreted the counted quantity based on the PLATO scale. Please see the revision on page 22, line numbers 504-513.

**Comment 8**: •NVIVO was used for qualitative analysis, but the coding framework is not provided. What themes were pre-identified? Were the codes data-driven or theory-driven?

**Response**: Thank you for an invaluable idea. We strongly agree with this comment and amended the manuscript to increase its quality, which is indicated on page 27, line numbers “618-637.”

**Statistical Problems**:

**Comment 9**: •**Regression models** lack justification for **predictor selection**.

**Response**: Yes, dear reviewer, it sounds nice. It would have been better if justified during submission, but we did consider it in this way. Thank you for this insight. Hence, we seriously amended our understanding of its importance. This idea was indicated in line numbers “593-604” on page 26. Accordingly, one theme was removed through the predictor section procedure. Thank you so much again.

**Comment** **10**: •VIF values are reported, but not interpreted correctly. The manuscript does not discuss potential collinearity issues in explanatory variables.

**Response**: Yes, we agree that it was not interpreted. As per your insight, we made a change. Please see line numbers “491-497”. The suggested reference was also incorporated into the manuscript as it was significant in indicating empirical insights into student learning challenges, essential for contextualizing the findings**.**

**Findings and Discussion (Pages 10-14)**

•Mismatch between Quantitative and Qualitative Findings:

**Comment** **11**: •Table 3 reports high instructor self-ratings (mean values around 3.7-4.0), yet qualitative findings suggest poor implementation.

**Comment** **12**: •There is no attempt to reconcile this contradiction—why do students and teachers perceive instructional practices so differently?

**Response**: Thank you so much. We responded to these comments by merging. These differences should have been discussed during the first submission. Your comment helped us to revise, so we have incorporated in limitation and suggested further recommendations that current researchers have not covered, and included in the discussion section the reason for the mismatch, though it was not enough which initiate the researchers to show limitations.

**•Statistical Errors:**
**Comment 13:** •The adjusted R² value of 0.288 in the regression model suggests that only 28.8% of the variance is explained. This is weak. What other variables might contribute to implementation differences?

**Response**: Thank you, reviewer. This appeared weak, or in the case of lower R-square values, we can consider confidence intervals and VIF, which could balance this problem, though we haven’t used. VIF in our findings showed a good standard, but still needs a strong R-Squared value (line “494”, p.21). Thus, the limitation for these issues you have raised (see limitation section L955-956, p.44).

**Comment 14:** •No effect sizes are reported. Effect sizes (Cohen’s d, **eta** squared) are critical in educational research.

**Response**: We appreciate your comment. We understand that the Beta value seemed enough in the regression model. Like your concern, some studies urge its significance in indicating a magnitude influence. This appeared in our studies and suggested for further research (see line numbers “957-959”, p.44).

•**Discussion Fails to Engage with Prior Studies:**

**Comment 15:** •There is no meaningful comparison with existing research. The discussion simply repeats the results without analyzing how they align with or challenge previous work.

**Response**: Thank you. Based on your insight, we made changes in the discussion section. Indicated interpretations and cited current literature as suggested (see line numbers “873-878”, pp. 40-41). The implication challenge was referred to the mismatch (see line numbers “830-832”, p.39).

•**Limitations Section is Underdeveloped:**

**Comment 16:** •The manuscript vaguely states, “instructors were theoretically grounded, but not reflected in practical application.” This needs quantifiable evidence. •The study’s geographical limitation to two Ethiopian universities is not adequately acknowledged as a constraint to generalizability.

**Response**: Thank you. The first question was revised in the text of the manuscript, and the second was included in the limitation section as it should be done as you commented. (See line numbers “960-961”, p.44). Limitations was also included in this regard (see line numbers “1010-1012”, p.46).

• **Recommendations are Not Practical:**
**Comment 17:** •The study suggests curriculum reform, but fails to outline specific pedagogical changes. How should universities modify teaching strategies?

**Response**: Thank you so much. We have revised the recommendation section thoroughly because it was not comprehensive as you suggested.

NB: Specific Recommendations for Major Revisions indicated in 1-6 were included in the response (comments 1-17).

**Comment 18:** Incorporate recent literature. Include the suggested references above.

**Response**: All newly cited references were incorporated into the manuscript. Some not recent but cited for purpose of importance. Indicated as follows.

**Newly incorporated References**

Lee JS, Chen Hsieh J. University students’ perceptions of English as an International Language (EIL) in Taiwan and South Korea. Journal of Multilingual and Multicultural Development. 2018 Oct 21; 39(9):789-802.

Luczaj K, Leonowicz-Bukala I, Kurek-Ochmanska O. English as a lingua franca? The limits of everyday English-language communication in Polish academia. English for Specific Purposes. 2022 Apr 1; 66:3-16.

Al Fraidan A. Evaluating lexical competency in Saudi Arabia's hybridized EFL ecosystem: A taxonomic exploration of vocabulary assessment modalities. International Journal of Distance Education Technologies (IJDET). 2025 Jan 1; 23 (1):1-36.

Al Fraidan A, Fakhli I. Vocabulary learning strategies of university students: The case of preparatory year students and English major students. Porta Linguarum. 2024 January; 2024 (41): 45-63.

Al Fraidan A. The impact of students’ misperceptions on test performance: A 3p model and self-determination theory approach. Edelweiss Applied Science and Technology. 2024; 8(5):1773-84.

O’Connor C, Joffe H. Intercoder reliability in qualitative research: Debates and practical guidelines. International journal of qualitative methods. 2020; 19 (1-13): 1-13. Available from: <https://doi.org/10.1177/1609406919899220>

Díaz J, Pérez J, Gallardo C, González-Prieto Á. Applying inter-rater reliability and agreement in collaborative grounded theory studies in software engineering. Journal of Systems and Software. 2023 Jan 1; 195: 111520.

Hemmler VL, Kenney AW, Langley SD, Callahan CM, Gubbins EJ, Holder S. Beyond a coefficient: An interactive process for achieving inter-rater consistency in qualitative coding. Qualitative Research. 2022 Apr; 22(2):194-219.

Hua M, Wang L. The relationship between Chinese university students’ learning preparation and learning achievement within the EFL blended teaching context in the COVID-19 post-epidemic era: The mediating effect of learning methods. PLOS ONE. 2023 Jan 24; 18(1):e0280919.

Goh CC, Vandergrift L. Teaching and learning second language listening: Metacognition in action. Routledge; 2021 Jul 27.

Chou MH. Strategies for interactive listening in modern foreign language learning. Language awareness. 2023 Jan 2; 32(1):169-91. Available from: https://doi.org/10.1080/09658416.2022.2047194

Komol T, Suwanphathama S. Students’ perceptions and attitudes toward the use of communicative language teaching (CLT) to improve English listening and speaking skills. English Language and Literature Studies. 2020; 10(4):40.

Sadeghi K, Bakhshi N. Integrated listening/speaking skill assessment: The role of ambiguity tolerance, cognitive/metacognitive strategy use, and foreign language anxiety. International Journal of Language Testing. 2025 Mar 1; 15(1):77-90.

Kang HY, Lee CH. Effects of focus on form instruction through listening in blended learning on the development of grammar and listening skills. 영어학. 2020 Mar; 20:662-91.

Ho S, Foulsham T, Kingstone A. Speaking and listening with the eyes: Gaze signaling during dyadic interactions. PLOS ONE. 2015 Aug 26; 10(8):e0136905.

Yamane T. Statistics: An introductory analysis. New York: Harper and Row, 2nd ed., 1973.

Illowsky B, Dean S. Introductory Statistics 2e. OpenStax; 2013 Sep 19.

Simeon M. Geographic information system and remote sensing based malaria risk mapping using environmental factors: A Case of Arba Minch Zuria Woreda, Southern Nations Nationalities and Peoples’ Regional State. Unpublished MSc thesis, Addis Ababa University, Ethiopia. 2014 Jun.

Hayidso TH, Gemeda DO, Abraham AM. Identifying road traffic accident hotspots areas using GIS in Ethiopia: a case study of Hosanna Town. Transport and Telecommunication. 2019; 20(2):123-32.

Yin RK. Qualitative research from start to finish. Guilford publications; 2015 Aug 5.

Langley A, Meziani N. Making interviews meaningful. The Journal of Applied Behavioral Science. 2020 Sep; 56(3):370-91.

Adhikari GP. Calculating the sample size in quantitative studies. Scholars' Journal. 2021 Dec 31:14-29.

Timotewos MT, Barjenbruch M, Behailu BM. The assessment of climate variables and geographical distribution on residential drinking water demand in Ethiopia. Water. 2022 May 27; 14(11):1722.

Lolaso T, Assef E, Woldeamanuel T. Impact of climate change on food security of smallholder farmers in Shashogo district, central Ethiopia. Climate Services. 2024 Apr 1; 34:100465.

Gehman J, Glaser VL, Eisenhardt KM, Gioia D, Langley A, Corley KG. Finding theory–method fit: A comparison of three qualitative approaches to theory building. Journal of Management Inquiry. 2018 Jul; 27(3):284-300.

Saldaña J. Coding techniques for quantitative and mixed data. The Routledge reviewer’s guide to mixed methods analysis. 2021 Jul 12:151-60.

Skjott-Linneberg M, Korsgaard S. Coding qualitative data: A synthesis guiding the novice. Qualitative research journal. 2019 Jun 4; 19(3):259-70.

Gioia DA, Corley KG, Hamilton AL. Seeking qualitative rigor in inductive research: Notes on the Gioia methodology. Organizational research methods. 2013 Jan; 16(1):15-31.

Thornberg R, Charmaz K. Grounded theory and theoretical coding. The SAGE handbook of qualitative data analysis. 2014; 5(2014):153-69.

Ryu N, Kim H, Kang P. Evaluating variable selection techniques for multivariate linear regression. Journal of Korean Institute of Industrial Engineers. 2016; 42(5):314-26.

Sestelo M, Villanueva NM, Roca-Pardiñas J. FWDselect: Variable selection algorithm in regression models. Discussion Papers in Statistics and Operations Research. University of Vigo, Vigo. 2013.

De Lozzo M, Marrel A. New improvements in the use of dependence measures for sensitivity analysis and screening. Journal of Statistical Computation and Simulation. 2016 Oct 12; 86(15):3038-58.

Akinwande MO, Dikko HG, Samson A. Variance inflation factor: As a condition for the inclusion of suppressor variable (s) in regression analysis. Open journal of statistics. 2015; 5(07):754.

Plonsky L, Ghanbar H. Multiple regression in L2 research: A methodological synthesis and guide to interpreting R2 values. The Modern Language Journal. 2018 Dec; 102(4):713-31.

Zaarour N, Melachrinoudis E. What’s in a Coefficient? The “Not so Simple” Interpretation of R^2^, for Relatively Small Sample Sizes. Journal of Education and Training Studies. 2019 Dec; 7(12).

Richardson JT. Eta squared and partial eta squared as measures of effect size in educational research. Educational research review. 2011 Jan 1; 6(2):135-47.

Yallew AT, Langa PV, Nkhoma N. English language in African higher education: A systematic review. Southern African Linguistics and Applied Language Studies. 2021 Jan 2; 39(1):5-29.

Burns A, Siegel J. Teaching listening: Dichotomies, choices and practices. In Handbook of practical second language teaching and learning. 2022 Sep; 29. 225-237. Routledge.

Chemir S, Kitila T. Learners’ needs analysis for English for academic purposes in Ethiopian higher education institutions: The case of Wachemo University freshman students. Cogent Education. 2022 Dec 31; 9(1):2026190.

Kabir SA. Policy and practice of listening in secondary English education: A critical reflection from Bangladesh. MEXTESOL Journal. 2024; 48(3):n3.

Chali KK, Parapatics A. Language policy and practices in an Ethiopian University towards multilingualism. Languages. 2024 May 28; 9(6):198.

Murphy AC, Mengistu MS. The challenges of internationalization in EMI tertiary education in Ethiopia. English-medium instruction and the internationalization of universities. 2020:77-101.

Simie T, McKinley J. English medium instruction in Ethiopian university mission statements and language policies. Language Policy. 2024 Feb 28:1-22.

Newton JM, Nation IS. Teaching ESL/EFL listening and speaking. Routledge; 2020 Oct 26.

Rahman MN, Chowdhury S, Mazgutova D. Rethinking teaching listening skills: A case study. Language Teaching Research Quarterly. 2023; 33:176-90.

Rost M, Brown S. Second language listening. In Handbook of practical second language teaching and learning. 2022 Sep; 29: 238-255). Routledge.

Terzioğlu Y, Kurt M. Elevating English language learners’ speaking fluency and listening skills through a learning management system. Sage Open. 2022 May; 12(2):21582440221099937.

Luo Y, Song H, Wan L, Zhang X. The effect of vocabulary depth and breadth on English listening comprehension can depend on how comprehension is measured. Frontiers in Psychology. 2021 May 25; 12:657573.

Ahmadnattaj F, Ostovar-Namaghi SA. On the Effectiveness of Integrated Skills Approach in Language Teaching: A Meta-analysis. Iranian Journal of Applied Linguistics. 2020 Sep 1; 23(2).

Dincer A, Dariyemez T. Proficient speakers of English as a foreign language: A focus-group study. IAFOR Journal of Education. 2020; 8(1):83-99.

Nguyen NH, Huynh NT. The Effects of integrating listening and speaking skills into Moodle-Based activities. In Proceedings of the Asia CALL International Conference 2021 Mar 1 (Vol. 533, No. 978-94-6239-343-1, pp. 166-174).

Sah FM, Shah PM. Teachers’ beliefs and practices in teaching listening. Creative Education. 2020 Feb 11; 11(2):182-95.

Ironsi CS. Improving communicative competence levels of pre-service teachers through spoken-based reflection instruction. Journal of Education. 2023 Jul; 203(3):678-89.

Hadi MS, Izzah L, Masae M. Factors affecting speaking anxiety of Thai students during oral presentation: Faculty of Education in TSAI. English Language in Focus (ELIF). 2020 Aug 29; 3(1):79-88.

Renandya WA, Hu G. L2 listening in China: An examination of current practice. International perspectives on teaching the four skills in ELT: Listening, speaking, reading, writing. 2018:37-50.

Becker A. Post-collection data capture, scoring, and processing. Reliability and Validity of International Large-Scale Assessment: Understanding IEA’s Comparative Studies of Student Achievement. 2020:151-67.
