# Supplementary material for: Investigating university English as a foreign language instructors’ implementations in teaching integral listening with speaking
Source: PLoS One. 2025 Aug 8;20(8):e0327029. doi: 10.1371/journal.pone.0327029 (PMC12334060; doi:10.1371/journal.pone.0327029)
Supplement: Attachment — Submitted filename: Response to the Reviewer 1_PHONE R2.docx [file pone.0327029.s009.docx]

**Date: 26 May 2025**

**Journal name: PLOS ONE**

**Article type: Original Research Article**

**Subject: The Response to Reviewer 1 Comments**

Dear Reviewer! My respectful greetings go to you!

I, the corresponding author of the Manuscript entitled “Investigating University English as a Foreign Language Instructor's Implementations in Teaching Integral Listening with Speaking” (**PONE-D-24-53961R1)**, would like to appreciate your constructive feedback and the valuable time you devoted to improving our work for consideration for publication in PLOS ONE. Your unreserved comment to review this manuscript uncovered our weaknesses in writing a manuscript for a journal like PLOS ONE. This encouraged the authors of this manuscript to revise it thoroughly.

Therefore, to ensure a well-structured appearance and readability of the Manuscript with the aforementioned title, the authors have illustrated the details, followed by a point-by-point response to your comments. The track changes made using the yellow highlight our responses.

Sincerely!

The corresponding author

**Point-by-Point Response to Reviewer #1 _PONE-D-24-53961R1**

**Comment 1**. There is a significant mismatch between instructors’ self-reports and students’ feedback, suggesting possible overestimation in instructor perceptions. While this discrepancy is acknowledged in the discussion, it would benefit from deeper triangulation with additional measures, such as rubric-based performance evaluations or third-party assessments.

**Response**: Our sincere gratitude and respect go to you, and we agree with your idea. It is believed that the rubric measures ensure that **all evaluators use the same standards,** improving the **reliability** and **objectivity** of the performance judgment. However, the present study used a single observer and interviewer, believing it appeared no need for other evaluators, but later we accepted it was our weakness. Let’s clarify some actions taken that might minimize this problem. To compensate for this gap, the present study involved multiple methods, such as quantitative and qualitative approaches employing a survey to quantify the data, interviews, and observations to qualify the data. Similarly, the study utilizes a different source population (teacher and student participants) to study the practice of integral listening and speaking, which could boost the validity and trustworthiness of the findings. Further, the study acknowledged this as a limitation and recommended it for future research (see p.47, lines 1038-1042, and track changed using yellow, and see p.45, lines 991-993, previously recommended).

**Comment 2.** The conclusions are generally consistent with the data, but should be framed more cautiously due to the descriptive and cross-sectional nature of the research design.

**Response**: The authors are grateful and appreciate your genuine concern about caution in conclusion, because it is an important section to attract the readers’ attention. Therefore, we revised based on your comment. You can refer to responses and track changes with yellow on page 44, line numbers 957-967.

**Comment 3**. The regression model has limited explanatory power (Adjusted R² = 0.288), and this should be more critically discussed in terms of implications.

**Response**: Yes, it is substantial comment to improve manuscript’s quality. We have discussed in terms of implications for language teaching learning in EFL contexts (see pp.49-50, lines 1097-1105), in addition to highlighting the limitations (see p.48, lines 1052-1064) and recommendations for further studies in a similar area of the study (see pp.45-46, line numbers 998-1002).

**Comment 4.** No inferential group comparisons (e.g., t-tests or ANOVA) were performed to examine differences across groups, which would have strengthened the analysis.

**Response**: Thank you for your valuable comments in this regard. Accordingly, we have revised the manuscript, incorporating an additional table indicating “Table 8: Inferential group comparison of practice” followed by analysis (see pp.32-33, line numbers, 717-725).

**Comment 5**. Effect sizes (Cohen’s d, eta squared) were not reported, limiting interpretation of practical significance.

**Response**: Thank you so much for the issue you forwarded, which is the foundation for the variables' explanatory power. Consequently, we have made changes and reported the eta-squared value and Cohen’s d value. Based on your enforcement, we understood that it shouldn’t have been reported. Accordingly, we have improved the manuscript (see p.33, lines 726-736). Moreover, the conclusion was strengthened (see p. 44, lines 957-965), recommendations (p.46, lines 1001-116), and implementations (p.50, lines 1103-1105) were incorporated in line with the conclusion.

**Comment 6**. The selection of regression predictors was not well justified initially; this was later addressed, but could still benefit from clearer theoretical alignment.

**Response**: We are grateful to have this question, as it is noteworthy to shape the article. Thus, our clarification was based on previous literature as indicated on page 39, lines 838-846. It is tracked with yellow.

**Comment 7.** Despite the authors’ statement that "all relevant data are within the manuscript and supporting information files," the underlying data are not fully available:

- No anonymized raw data from surveys (Likert responses) are included.
- No SPSS or regression output files are provided.
- The NVivo-coded qualitative data or coding frameworks are not attached.
- The data are not deposited in any public repository, which is preferred under PLOS ONE’s data policy.

The authors must upload all underlying data (survey responses, regression model variables, and qualitative coding schema) to a recognized open-access repository (e.g., Zenodo, Figshare) or provide them as supplementary files.

**Response**: Thank you for your reminder. In the previous submission, we tried to upload such data, but the raw data of survey responses were not included. Now, as per your comments, we have uploaded anonymized raw data from surveys (Likert responses), regression output files, and the NVivo-coded qualitative data, as initial codes or seven children's codes were derived, followed by the aggregate codes. Similarly, to deposit the data as a public repository, which is preferred under PLOS ONE’s data policy, we uploaded it as supplementary material independently (like survey responses, regression model variables, and qualitative coding) in addition to supplementary materials related to tools of data collection, including interview transcription.

Additionally, to abide by the PLOS ONE Open Access Policy, while revising our submission, we have uploaded figure files to the Preflight Analysis and Conversion Engine (PACE) digital diagnostic tool.

**Comment 8**. The revised version has improved in clarity and readability. However, a few sections—particularly in the methodology and discussion—would benefit from tighter organization and more concise phrasing. Please ensure final proofreading for grammatical consistency.

**Response**: The methodology and discussion were improved accordingly. Finally, we have improved the language thoroughly for grammatical consistency and accuracy. This was assisted by the Grammarly software in addition to line-by-line proofreading.

All recommendations were addressed through the above specific comments (1-8).

**Overall, thank you in advance for your unreserved contribution to shaping our work!**
